# Transmission Eigenvalues in Chiral Molecular Junctions

**DOI:** 10.1021/acsomega.6c05035

**Published:** 2026-07-06

**Authors:** Ulrich Pototschnig, Sumit Naskar, Thorsten Hansen, Carmen Herrmann

**Affiliations:** † Department of Chemistry, 14915 Universität Hamburg, HARBOR Bldg. 610, Luruper Chaussee 149, 22761 Hamburg, Germany; ‡ The Hamburg Centre of Ultrafast Imaging, Luruper Chaussee 149, 22761 Hamburg, Hamburg, Germany; § Department of Chemistry, University of Copenhagen, DK 2100 Copenhagen, Denmark

## Abstract

Chiral-induced spin selectivity (CISS) is caused by the interplay between the chirality of a system and electron magnetic moments in nonequilibrium. CISS manifests itself in two-terminal junctions (e.g., electrode–molecule(s)–electrode) in magnetoresistance. For a given magnetization orientation of the ferromagnetic electrode, l- and d-enantiomers show different current–voltage behavior. Reversing the magnetization reverses the current response of l and d, respectively. The fact that this happens near zero bias in the linear regime has prompted a discussion on the consequences of time-reversal symmetry for the possible mechanisms underlying CISS, which are often based on simplifying the junction to an effectively one-dimensional system or to an idealized helix with two orbitals per site. This motivates us to explore the electron transport characteristics of realistic chiral molecular junctions as typically studied in experiments. As a measure of how strongly the junctions deviate from idealized cases, we focus on the number of nonzero transmission eigenvalues. We find that all systems considered exhibit at least two transmission eigenvalues that lie clearly above the noise threshold. This is most pronounced for unsubstituted [6]-helicene, thio-[6]-helicene, [6]-carboxyhelicene, and a pentapeptide α-helix with thiolated terminal groups and less pronounced for helicenes with amine, bromine, or thiadiazole anchoring groups and for short linear peptides. We therefore propose a comparative study of molecules with differently pronounced second transmission eigenvalues, under identical experimental conditions, to assess the relevance of these transmission eigenvalues for the CISS effect.

## Graphical abstract

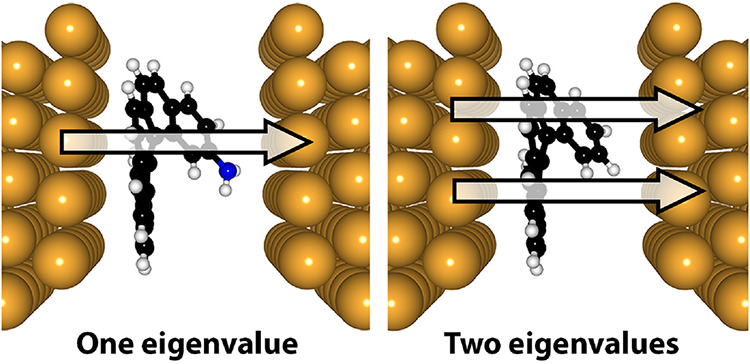

## 1 Introduction

Interactions between electron spins, and chiral molecules and materials have been thoroughly investigated in recent years. Electrons propagating through a chiral medium are transmitted with different probabilities depending on the orientation of their magnetic moment vector and on the handedness of the medium, when an external magnetic field is absent. This effect is known as chiral-induced spin selectivity (CISS).^1–4^ CISS is promising for applications in spintronics, catalysis, and biological processes, and it may help answer questions regarding the origin of life.^5–10^ There are several experimental techniques to observe the CISS effect in chiral molecules and materials.^3,9,11,12^ Most notably, experimental studies on CISS have demonstrated the occurrence of CISS in two-terminal junctions, usually containing a monolayer of chiral molecules, where one electrode is ferromagnetic, such as a magnetic conductive-probe atomic force microscopy or a spin-polarized scanning-tunneling microscopy setup.^13–15^ Similar results have also been documented for chiral single-molecule junctions^11,16^ (although for such cases, also negative results have been reported^17^). A schematic of the characteristic current–voltage behavior of CISS is shown in Figure 1. For a pair of enantiomers l and d, the current response is enantiomer- and magnetization-dependent in such a way that *I*_L_(+*M*) = *I*_D_(−*M*) and *I*_D_(+*M*) = *I*_L_(−*M*), if the magnetization direction (*M*) of the ferromagnetic electrode is fixed either parallel (+) or antiparallel (−) w.r.t. electron transport direction. The normalized difference between the parallel and antiparallel currents is referred to as CISS magnetoresistance (CISS-MR).

**Figure 1:**
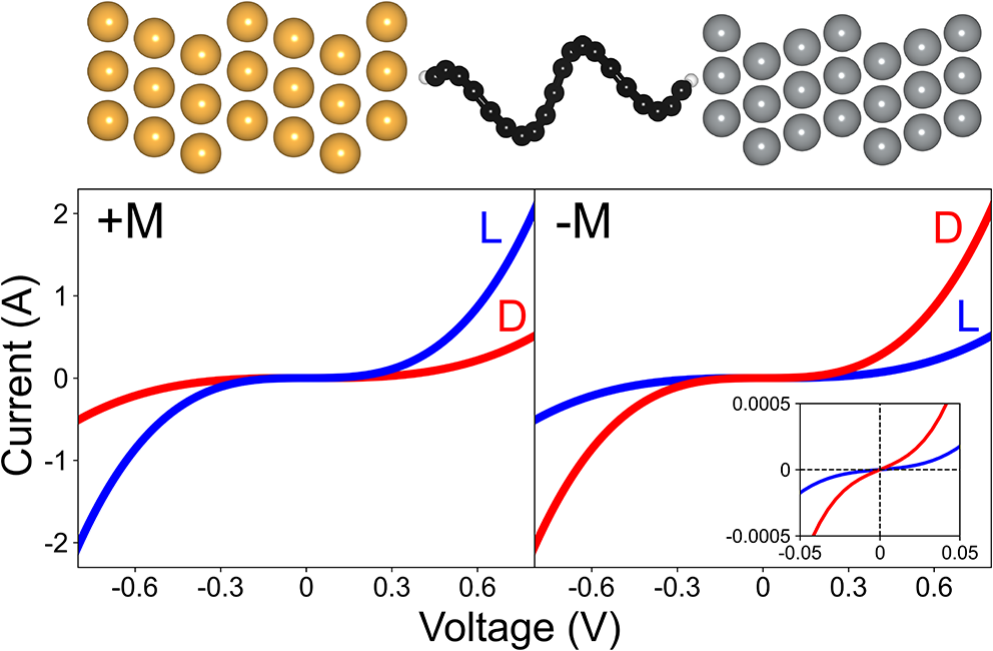
Schematic representation of a two-terminal molecular junction containing a nonmagnetic electrode, a chiral molecule (illustrated here with a fictional carbon helix), and a ferromagnetic electrode (top), and of a typical CISS *I*-*V*-curve (bottom): The current response of S- and d-enantiomers is reversed upon electrode magnetization (±M) reversal. The difference in zero-bias conductance is emphasized in the inset.

It is important to note that CISS-MR is distinct from spin polarization, as it does not involve a direct measurement of electron spin.^18,19^ This magnetoresistive behavior is also present close to zero bias, which is usually considered the linear regime, as indicated by the inset of Figure 1. However, this appears to violate Onsager’s reciprocal relations.^20,21^ Onsager’s reciprocal relations imply that close to equilibrium and in the case of diffusive electron transport, linear-response coefficients, such as conductance, must be symmetric under reversal of time-reversal-odd parameters, such as magnetic field *B* or magnetization *M*. This is due to the fundamental principle of microscopic reversibility, which is a consequence of time-reversal symmetry.^22^ Several possible solutions to this (apparent) contradiction between experiment and theory have been proposed such as a modification of the transport barrier by the magnetization direction^23^ or the general questioning of the Onsager reciprocal relationship for nondiffusive forms of charge transport through chiral molecules, such as tunneling.^24^ While the latter argument suggests that the Onsager relations may not apply to CISS magnetoresistance experiments, the underlying reason why chiral systems would fail to obey Onsager reciprocity remains unclear, particularly given that reciprocity has been demonstrated in tunneling-based transport experiments.^25^

It has also been suggested that the transmission eigenvalues for a two-terminal system with half-integer spin occur in degenerate pairs (comprising the two spin orientations for a given transport direction) due to time-reversal symmetry^26^ (Bardarson’s theorem). As proposed by Utsumi et al.,^27,28^ a junction containing two spatially distinct transport channels, e.g., made up by the *p*_*x*_ vs *p*_*z*_ orbitals in a helical tight-binding chain (and mixed by spin–orbit coupling), yields two pairs of degenerate transmission eigenvalues. This degeneracy is required by time-reversal symmetry and is fully consistent with Bardarson’s theorem. Within these degenerate pairs, spin flips can correlate with flips of the orbital channels; thus, one pair could be associated with two up spins propagating in one direction, while the other pair could correspond to two down spins propagating in the opposite direction. As a result, this could lead to spin-polarized currents in the linear regime even in a two-terminal, time-reversal-symmetric setup. Importantly, such spin polarization does not imply a finite linear-response magnetoresistance in a chiral molecular junction.^20^ However, we want to use this aspect as a motivation to investigate how many significant transmission eigenvalues are present in realistic models of two-terminal chiral molecular junctions. In contrast to previous work, we will model these with first-principles electronic structure methods, containing their full atomistic complexity, and explicitly including the atomistic structure of the electrodes (which we both model as gold, as magnetic electrodes such as nickel are usually covered by a few-nanometer gold layer in experiments).

## 2 Background: Transmission Eigenvalues

The definition of channels in the context of electron transport and their relation with transmission eigenvalues are not always clear in the literature, in particular when dealing with realistic atomistic models of a nanoscale transport junction. Without any claim to being exhaustive, we review in the following some instances where these concepts have been addressed and discuss their relation to the terminology used in this work.

A channel, also often referred to as a “mode”, is often understood as an independent (additive) pathway for electron propagation.^29^ The existence of such channels has been linked to experimental observations of quantized conductance steps of point contacts in a two-dimensional electron gas, where each conductance step corresponds to the opening of a new channel.^30,31^ The conductance *G* in a two-terminal junction of point contacts in a two-dimensional electron gas is then

1 $$G=\frac{2e^{2}}{h}N_{C}$$

where *e* is the elementary charge, *h* is the Planck constant, and *N_C_* is the number of (in this case fully open) channels. For single atoms, the number of channels corresponds to the atom’s valence.^32,33^

CISS magnetoresistance experiments featuring chiral molecules fall into the category of two-terminal *molecular* junctions. There was an aspiration to identify a set of “natural” conductance channels in such molecular junctions characterized by symmetric generalized orbitals, understood to correspond to the bias-voltage-dependent spatially resolved scans observed in the scanning-tunneling microscope^34^ (which would reduce to molecular orbitals in the limit of zero molecule-electrode coupling). However, these idealized channels remained elusive, and no unique criterion for their identification has emerged. Most notably, Solomon et al. demonstrated that the nonzero transmission eigenvalues and, thus, the number of contributing channels varies when changing the definition of “channels”, with only certain definitions being compatible with the interpretation of shot-noise experiments.^35,36^ Bergfield et al. established a correlation between the number of dominant transmission eigenvalues and the degeneracy of the molecular orbital (MO) closest to the Fermi level.^37^ However, this cannot be viewed as a universal rule as the contribution of an MO to the zero-bias current is determined by its energy position w.r.t. *E*_F_, the degree of localization, as well as the coupling to the leads.^38^ Alternative definitions have been explored in the past, e.g., to improve the description of inelastic electron tunneling spectroscopy spectra^39–41^ or to separate transmission contributions from individual orbitals and orbital pairs.^35,42,43^

In first-principles calculations on molecular junctions, one often evaluates transmission matrix eigenvectors or so-called eigenchannels with their respective eigenvalues λ_*i*_, which add up to the total transmission *T*

2 $$T\left(E\right)=\sum_{i}\lambda_{i}\left(E\right)$$

and which depend on the energy *E* of the tunneling electron. Transmission is a quantity roughly corresponding to a tunnneling probability density and is often evaluated within the Landauer–Imry–Büttiker formalism combined with the nonequilibrium Green’s function (NEGF) approach.^44^ This approach assumes coherent electron tunneling between two semi-infinite leads (electrodes) through a two-terminal junction (molecule).^44,45^ It describes the conductance at zero bias *G*(0*V*) as

3 $$G\left(0V\right)=G_{0}T\left(E_{F}\right)=\frac{2e^{2}}{h}T\left(E_{F}\right)$$

where $G_{0}=\frac{2e^{2}}{h}$ is the quantum of conductance and *T*(*E*_F_) is the total transmission at the Fermi level *E*_F_.^46^ *T*(*E*) can be obtained by the trace of a transmission matrix ***T*** (*E*),

4 $$T\left(E\right)=\mathrm{Tr}\left[T\left(E\right)\right]=\mathrm{Tr}\left[\Gamma^{L}\left(E\right)G\left(E\right)\Gamma^{R}\left(E\right)G^{†}\left(E\right)\right]$$

which, in turn, can be obtained from the retarded Green’s function matrix ***G*** and the spectral densities **Γ** ^*L*^ and **Γ** ^*R*^ (see Section 3).

This energy-resolved expression for the transmission function *T*(*E*) obtained from our NEGF calculations relates to the transmission *T* appearing in Landauer’s original formulation based on scattering theory, where *G* = *G*_0_ *T*. There, the transmission was expressed as the sum over transmissions *T*_*ij*_ from a mode *j* in the left lead to a mode *i* in the right lead

5 $$T=\sum_{i,j=1}^{N}T_{\mathrm{ij}}=\sum_{i=1}^{N}T_{i}=\mathrm{Tr}\left[tt^{†}\right]$$

where ***t*** is the transition matrix from scattering theory, and *t*_*ij*_ gives the amplitude for transitioning from a mode *j* in the left lead to a mode *i* in the right lead.

The spectral densities are given as **Γ** ^*L*^ = 2π∑_*d*_ ***V*** ^2^δ­(*E* – *E*_*d*_) and **Γ** ^*R*^ = 2π ∑_*a*_ ***V*** ^2^δ­(*E* – *E*_*a*_), where ***V*** is the coupling between lead and molecule. We can insert this into eq 4 to obtain

6 T(E)=4π2Tr[VG(E)VVG†(E)V]×δ(E−Ed)δ(E−Ea)(6)=4π2∑adtda(E)2×δ(E−Ed)δ(E−Ea)(7)

where the transition matrix is given as ***t*** (*E*) = ***VG*** (*E*) ***V***. Now compare eqs 5 and 7.

## 3 Methodology

We performed Kohn–Sham density functional theory calculations to obtain the electronic structure of various two-terminal molecular junctions. Calculations were performed using the software package QuantumATK.^47^ (M)-[6]-Helicene between two Au electrodes was used as a test system, as seen in Figure 2. As already mentioned in Section 1, we use gold for both electrodes. This choice reflects the experimental situation: Although many CISS-MR experiments employ ferromagnetic Ni substrates, these are typically coated with a gold layer of several nm thickness. Because no ferromagnetic layer is present in our simulation setup, our study does not aim to assess whether linear-response CISS-MR can be captured within this model. Instead, we focus on analyzing the transmission eigenvalues of the junction. The Au electrodes with a (111) surface comprised 5 × 3 × 3 unit cells, therefore containing 225 atoms. The calculations were carried out under periodic boundary conditions. A density mesh cutoff of 200 hartree and a *k*-point grid of 6 × 6 × 53 were found to be optimal and used for every calculation, with the latter grid in the transport direction. Every electronic structure calculation was carried out at a bias of 0 V.

**Figure 2:**
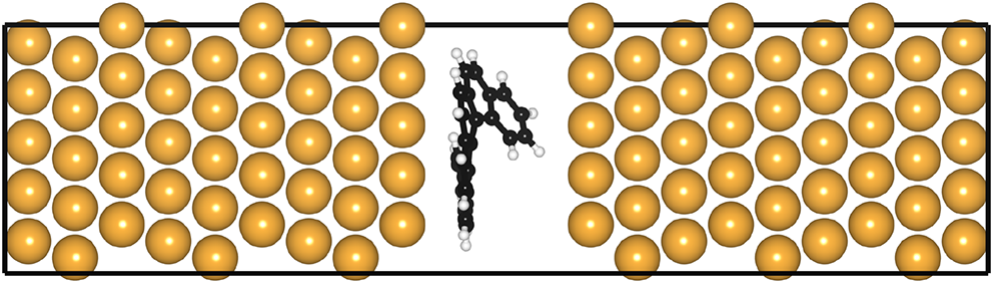
Molecular junction of (M)-[6]-Helicene between two Au electrodes. Note that in CISS experiments of the magnetoresistance type, one of the electrodes is magnetic (often Nickel). Due to it being typically coated with a few-nm gold layer, we model our junctions as having two gold electrodes.

In principle, the electron density can be computed either by summing over the occupied scattering states or via the NEGF formalism. In practice, NEGF is the standard approach in first-principles conductance calculations due to its common integration with methods such as density functional theory.^47^ Within the NEGF method, the two-terminal junction is divided into (a) a central region, which contains the molecule and, often, several adjacent electrode layers, and (b) two electrodes, which are treated as semi-infinite periodic structures acting as reservoirs of electrons. We employ the NEGF method as implemented in QuantumATK, where total transmission *T* from eq 3 and transmission matrix ***T*** is calculated via eq 4, where ***G*** (*E*) is the retarded Green’s function matrix of the central region and **Γ** ^*X*^ (X = L, R) are the coupling (or broadening) functions of the respective electrodes,^42^ as implemented in QuantumATK. *T*_*nn*_(*E*) denotes the diagonal entries of the transmission matrix. The Green’s function matrix ***G*** (*E*) is calculated from the central region effective single-particle Hamiltonian matrix ***H*** (*E*), the overlap matrix ***S***, and the electrode self-energies **Σ** ^*X*^, which describe the effect of each semi-infinite electrode on the finite central region, via

8 $$G\left(E\right)=[\mathrm{ES}-H\left(E\right)-\Sigma^{L}\left(E\right)-\Sigma^{R}\left(E\right){]}^{-1}$$

For our purposes, we do not perform relativistic electronic structure calculations to obtain spin-dependent transport or CISS-MR. The evaluation of two-component transmission eigenvalues is not implemented at present in QuantumATK, and we also do not expect them to depend substantially on the inclusion of relativistic effects. As a sanity check, we performed a comparison between a nonrelativistic and two-component calculation of the transmission (Figure S3 in the Supporting Information). The transmission function is only shifted slightly in energy while the overall shape remains the same, suggesting that our approach of only considering nonrelativistic spin-free calculations to obtain transmission eigenvalues is justified. Furthermore, our conclusions depend only on the relative size of transmission eigenvalues, not on the magnitude or microscopic origin of the CISS magnetoresistance.

To account for the *k* dependence of transmission, eigenvalues were evaluated at the *k* point exhibiting the highest overall transmission at the Fermi level, ensuring strong contributions to the total transmission. The choice of *k*-point does not change the overall qualitative picture, see Section S2 for more details.

The influence of exchange–correlation functionals was evaluated for (M)-[6]-Helicene with different anchoring groups. The selection was based on whether the respective anchoring group on helicene has been previously investigated experimentally in the context of CISS or has been successfully synthesized to date. Six different anchoring groups were chosen. The respective molecular junctions are illustrated in Figure 3: hydrogen (−H),^16,48^ amine (−NH_2_),^49,50^ carboxyl (−COOH),^51^ bromine (−B), dithiol (−S),^52^ and thiadiazole (−C_2_H_2_N_2_S).^53,54^

**Figure 3:**
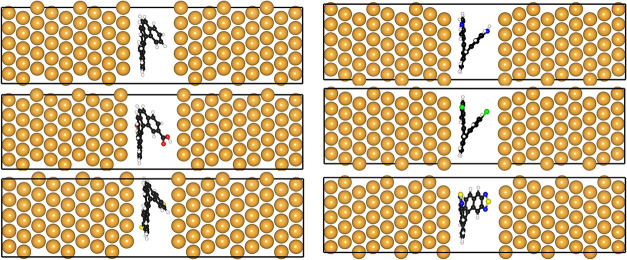
Illustration of investigated (M)-Helicenes with different anchoring groups adsorbed on Au in a molecular junction setup. From left to right, top: hydrogen (-H), and amine (−NH_2_) anchoring groups; middle: carboxyl (−COOH) and bromine (−B) anchoring groups; bottom: dithiol (-S) and thiadiazole (−C_2_H_2_N_2_S) anchoring groups.

The generalized gradient approximation functional by Perdew, Burke, and Ernzerhof (GGA-PBE)^55^ in combination with the PseudoDojo^56^ pseudopotential with a medium basis set size (see Section S1) was used for every molecular structure optimization. Each molecule was initially structurally optimized using the same junction setup and parameters as described above, but with only one electrode present. The atomistic structures were optimized using the L-BFGS algorithm with a force tolerance of 0.05 eV/atom.^57^ The resulting equilibrium configurations were subsequently used for a second atomistic structure optimization on a gold surface to mimic realistic structures in a two-terminal junction. This final structure was then used to construct molecular junctions. All atomistic structures and other raw data are provided as Supporting Information (see Section 8).

## 4 Results and Discussion

First, the influence of the exchange-correlation functionals PBE (GGA), *r* ^2^SCAN^58^ (meta-GGA), and HSE06 ^59–61^ (hybrid) as well as the anchoring group are examined for the case of helicene connected to gold electrodes. It is worth noting that helicene will not sit symmetrically between two electrodes, but adsorb primarily on one electrode as described in the previous section, in order to mimic a realistic two-terminal junction setup. The pseudopotential is set to a ‘medium’ level of PseudoDojo (see Section 3) as a balance between accuracy and computational demand. An evaluation of different pseudopotentials and basis sets can be found in Section S1.

Figure 4 presents the transmission eigenvalues for each configuration at *E*_F_. Both enantiomers were considered, where (P)-[6]-helicene was created by mirroring the relaxed adsorption structure of (M)-[6]-helicene on gold without mirroring the gold electrode. Even though a (111) gold surface is achiral, this was done to ensure that any change in chirality originated solely from the molecule. The results are shown in Figure 4. A normalization of the eigenvalues w.r.t. the primary eigenvalue was applied to improve visual clarity, as the absolute values of the transmission eigenvalues are not of great interest for our analysis but rather their relative magnitudes.

**Figure 4:**
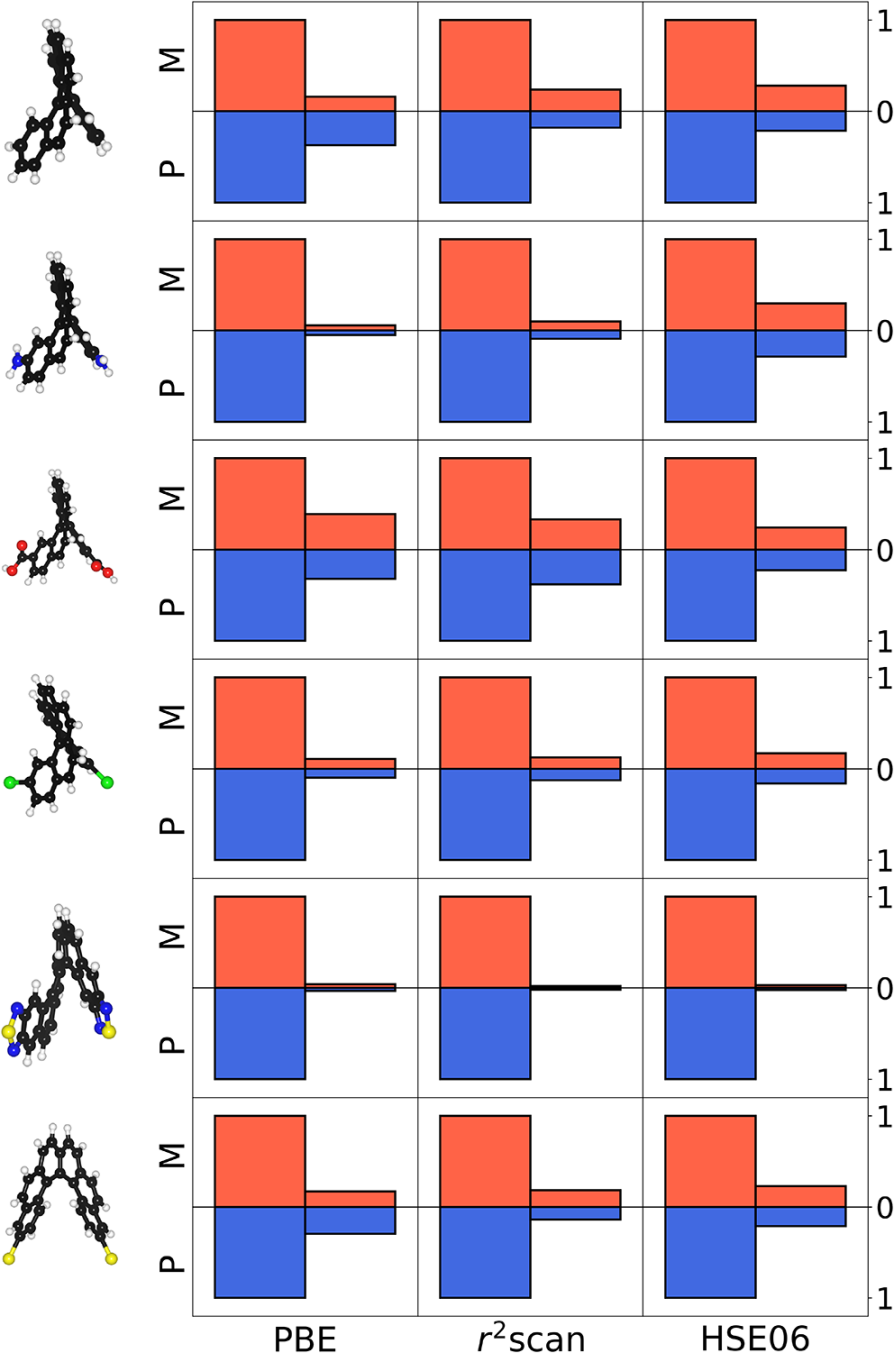
Transmission eigenvalues normalized w.r.t. the primary (largest) transmission eigenvalue for both (M)-[6]-Helicenes (red) and (P)-[6]-Helicenes (blue). The columns correspond to the three different exchange-correlation functionals PBE (GGA), *r* ^2^-Scan (meta-GGA) and HSE06 (hybrid), while the rows correspond to the six different “anchoring groups” hydrogen/unsubstituted (−H), amine (−NH_2_), carboxyl (−COOH), bromine (−B), thiadiazole (−C_2_H_2_N_2_S), and thiol (−S). The corresponding data set can be found in Section S5. Note that the ball-and-stick models illustrate the different functional groups and do not reflect the actually adsorbed molecular structures as used in the calculations.

Although each system contains at least two transmission eigenvalues that are clearly above the numerical noise level (see Section S5 for the raw data of the first four eigenvalues), the magnitudes depend strongly on the anchoring group. For the bromine-substituted helicene, the second transmission eigenvalue is just above 10% of the main eigenvalue. For amine anchoring, the answer depends strongly on the exchange–correlation functional, with HSE06 giving a larger second eigenvalue than PBE, which is consistent with previous experimental results.^62^ The thiol anchoring consistently shows a pronounced secondary transmission eigenvalue above 10%, contrary to an experimental eigenchannel study on Au–1,4-benzenedithiol–Au junctions.^63^ For the carboxyl-substituted helicene, the dependence on the functional is, interestingly, reversed—but here, all three suggest that there is a clear second eigenvalue. Overall, we can robustly state that each helicene derivative yields at least two transmission eigenvalues above the noise level.

Studying the impact of structural fluctuations on transmission eigenvalues is beyond the scope of this study, but the chirality-dependent variations seen in Figure 4 give a first indication of their influence. These variations may result from different relative orientations of the gold atoms with respect to the molecular structure. As previously mentioned, the second enantiomer was obtained by mirroring only the molecular structure, without mirroring the gold electrode (for more details, see Section S4 in the Supporting Information). This asymmetry leads to small variations in the distances between the gold atoms and the molecular atoms, even though the overall distance from the surface plane remains the same, potentially contributing to the observed differences. Both observed sensitivities to the Fermi level and the influence of atomistic structure have also been discussed in earlier work.^64^

To summarize, there can be significant differences in the eigenvalue magnitude between different exchange–correlation functionals, as seen, e.g., in the second structure in Figure 4, (M)-bis­(6-amino)-[6]-helicene. Moreover, there is also no systematic change in the magnitude when comparing the different functionals. Hence, the choice of functional adds to other uncertainties when transmission channels are meant to be evaluated quantitatively at *E*_F_, such as shortcomings in finding the correct Fermi level using DFT from an individual snapshot of a fluctuating molecular junction. We will use the hybrid functional HSE06 for the calculations in the following, but it should be kept in mind that the uncertainties just discussed add to an error bar.

We finally investigate further molecules which are experimentally relevant in the context of CISS; (L)/(D)-lysine and a glycine-based pentapeptide in the shape of a (l)/(d)-α-Helix with thiolated terminal groups, representative of various peptide-based CISS magnetoresistance experiments,^13,14,65,66^ (M)/(P)-[6]-Helicene,^11,16^ (3S)/(3R)-3-methylhexane-1,6-diol, (R/S)-2-methylbutanediamide^67^ and (l)/(d)-tartaric acid^68–70^ (Figure 5). The hybrid functional HSE06 and the pseudopotential set PseudoDojo (medium) were used with the same parameters as described above. The data can be found in Section S5. Despite substantially smaller secondary transmission eigenvalues for (l)/(d)-lysine as well as (l)/(d)-tartaric acid, all systems still exhibit eigenvalues that lie robustly above the noise level.

**Figure 5:**
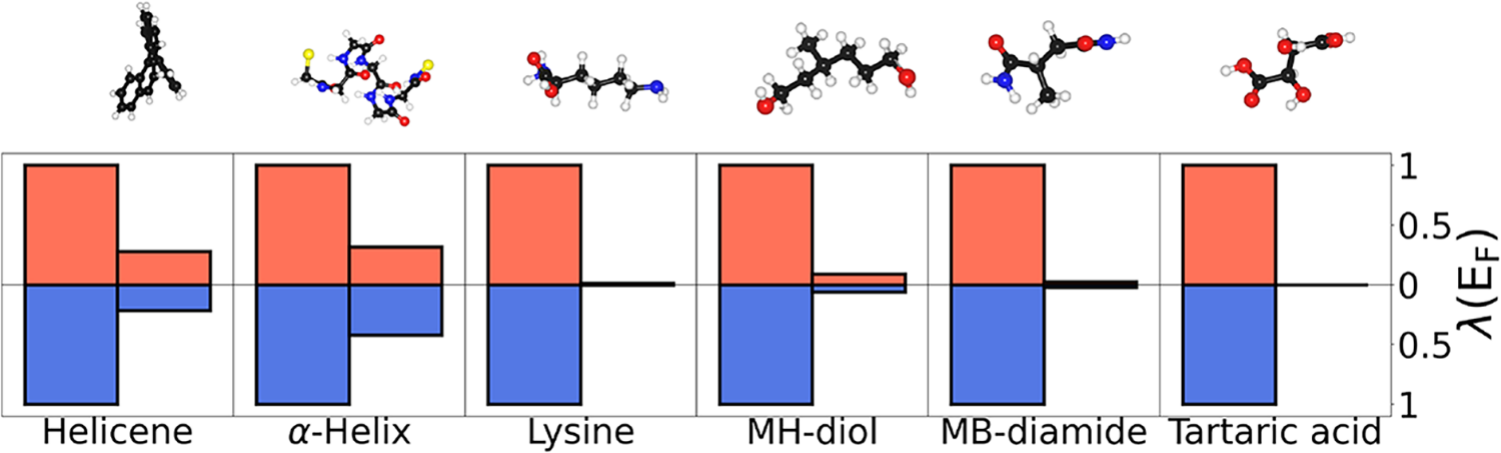
Transmission eigenvalues for various experimentally relevant left-handed (red) and right-handed (blue) chiral molecules. The hybrid functional HSE06 and the pseudopotential set PseudoDojo (medium) were used. The corresponding data set can be found in Section S5.

## 5 Conclusion

In summary, we calculated the transmission eigenvalues for a series of two-terminal junctions of various molecules using the Landauer/NEGF approach, combined with density functional theory. We examined the effects of the exchange-correlation functional, finding that while the uncertainty associated with its choice leads to an error bar, for many molecules, we can make a robust statement on whether there is a significant second transmission eigenvalue. Our calculations indicate that all systems considered exhibit at least two transmission eigenvalues that are clearly above the noise threshold, with the second eigenvalue being pronounced to varying degrees. This would suggest comparing these two sets of molecules in a CISS-MR experiment under identical conditions (possibly complemented by other probes sensitive to chirality and magnetism, such as electrical magnetochiral anisotropy (eMChA)^71^) to learn more about the significance of transmission eigenvalues for CISS, along with gaining insight into fundamental aspects related to the validity of simple models for evaluating the consequences of time-reversal symmetry.

## Supplementary Material



## Data Availability

The data sets generated and analyzed during this study are available in the Zenodo repository (DOI: 10.5281/zenodo.20721430).

## Notes

Influence of pseudopotentials; k dependence of transmission eigenvalues; influence of spin–orbit coupling; influence of mirroring the molecule; and data for Figures 4 and 5 in the main manuscript (PDF)

## ∥

Institute of Physical Chemistry, Friedrich-Schiller Universität Jena, 07743 Jena, Germany
