# Supplementary material for: Transmission Eigenvalues in Chiral Molecular Junctions
Source: ACS Omega. 2026 Jul 6;11(28):42893–900. doi: 10.1021/acsomega.6c05035 (PMC13393379; doi:10.1021/acsomega.6c05035)
Supplement: Supplementary file 1 [file ao6c05035_si_001.pdf]

Transmission Eigenvalues in Chiral Molecular Junctions - Supplementary Information

Ulrich Pototschnig,[†] Sumit Naskar[a],[†,‡] Thorsten Hansen,[¶] and Carmen Herrmann[*,†,‡]

†*Department of Chemistry, Universität Hamburg, HARBOR Bldg. 610, Luruper Chaussee 149, 22761 Hamburg, Germany*

‡*The Hamburg Centre of Ultrafast Imaging, Luruper Chaussee 149, 22761 Hamburg, Hamburg, Germany*

¶*Department of Chemistry, University of Copenhagen, DK 2100 Copenhagen, Denmark*

E-mail: carmen.herrmann@uni-hamburg.de

[a]Present address: Institute of Physical Chemistry, Friedrich-Schiller Universität Jena, 07743 Jena, Germany

S1 Influence of pseudopotentials

We examine the influence of pseudopotentials on the transmission channels of a (M)-[6]-Helicene junction with two gold electrodes as shown in Fig. 2, both at the HOMO energy level E_{HOMO} and Fermi level E_F. Four different pseudopotentials were considered: Optimized norm-conserving potentials SG15[1], PseudoDojo[2], a Hartwigsen-Goedecker-Hutter (HGH) type[3] and polarized single-ζ and double-ζ basis sets obtained from the FHI package[4]. Two different LCAO basis sets were used for each pseudopotential. The developers of QUANTUMATK thoroughly tested SG15 and PseudoDojo; thus, the term 'high' refers to a basis set necessary to obtain DFT

energies of certain test systems to change by no more than 1 meV/atom. For the ‘medium’ basis set, this constraint was relaxed to 4 meV/atom[5]. Results are shown in Fig. S1. Each system yields at least five channels. However, since many of these channels have very small eigenvalues ($<10^{-10}$), only the three largest eigenvalues are shown. Typically, one dominant channel is developed with one or two secondary channels which are usually negligible. While the relative channel contributions vary by approx. 40% at E_{HOMO}, the choice of pseudopotential can change the transmission eigenvalues by more than one order of magnitude at E_F, which illustrates the limitations of DFT in reliably predicting the Fermi energy. This is especially striking for the ζ-basis sets. Moreover, a larger basis set does not necessarily yield higher transmission values as can be seen for eigenvalues at E_{HOMO}. Nevertheless, a larger basis set should provide a more accurate representation of the electronic states within a system, and therefore, a more precise description of resonances, which is essential for an accurate evaluation of transmission coefficients.

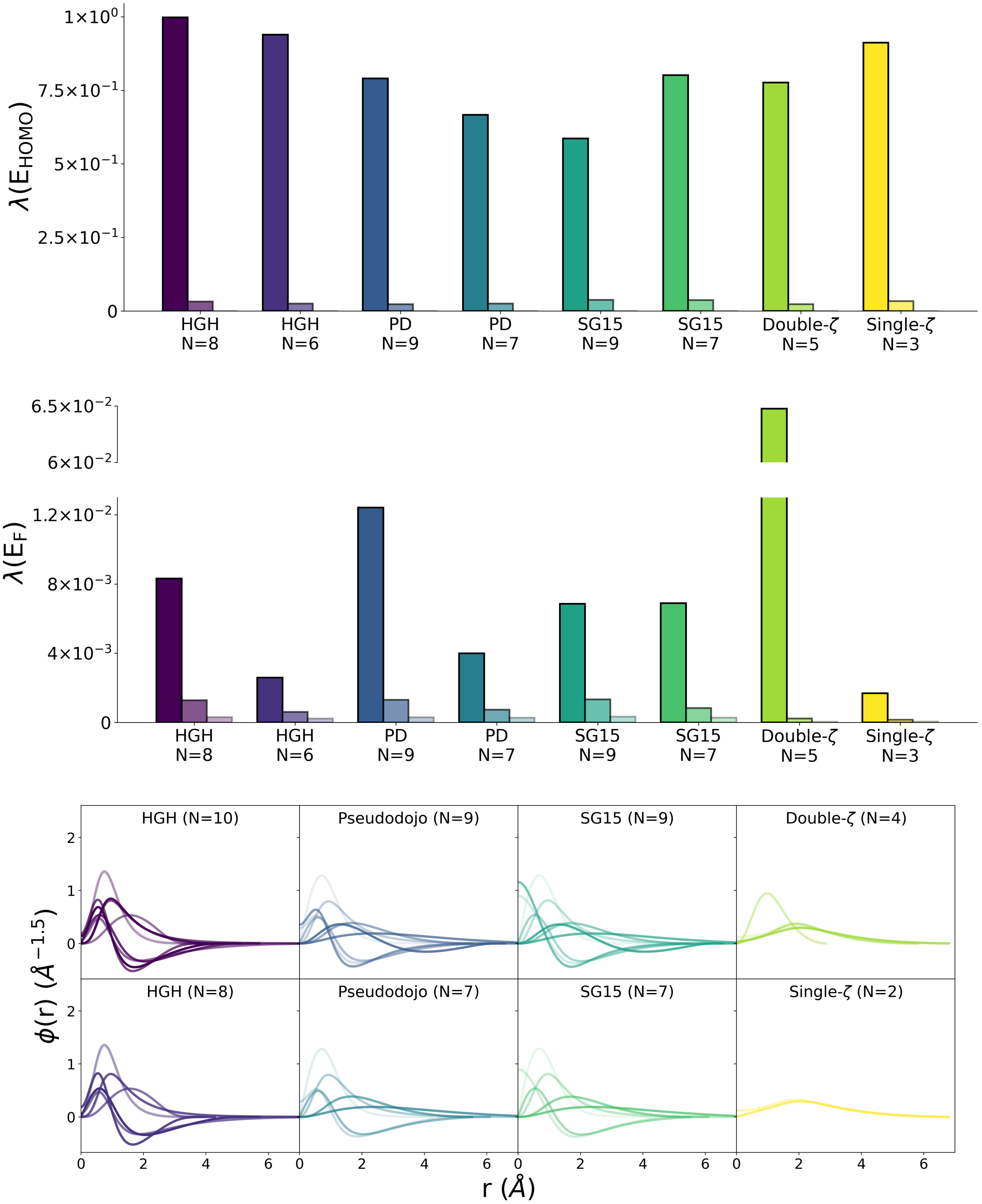

Figure S1: Transmission eigenvalues at E_{HOMO} (top) and E_F (middle) of (M)-[6]-Helicene between two Au electrodes for different pseudopotentials and basis set sizes (N). The basis function for each basis set are visualized in the bottom plot.

S2 k-dependence of transmission eigenvalues

To examine the k-dependence of the overall transmission and the transmission eigenvalues, we selected four systems for a detailed analysis. Specifically, (M)-[6]-thio-helicene, (3S)-3-methylhexane-1,6-diol, (S)-2-methyl-butanediamide and (L)-α-Helix with thiolated terminal groups were considered. Fig. S2 shows the k-dependence of the overall transmission. The transmission exhibits a maximum for three out of four test systems at at the Γ-point. Transmission eigenvalues were calculated on 25 points as indicated by the white dots. The right column of Fig. S2 shows the corresponding transmission eigenvalues. Lower overall transmission tends to exhibit a higher secondary transmission eigenvalue. However, and most notably, the transmission eigenvalues exhibit a consistent qualitative behavior, with every point in k-space displaying at least two significant eigenvalues. This analysis also validates our approach of selecting the k-point with the highest transmission, as it does not vary too strongly depending on the choice of k-point.

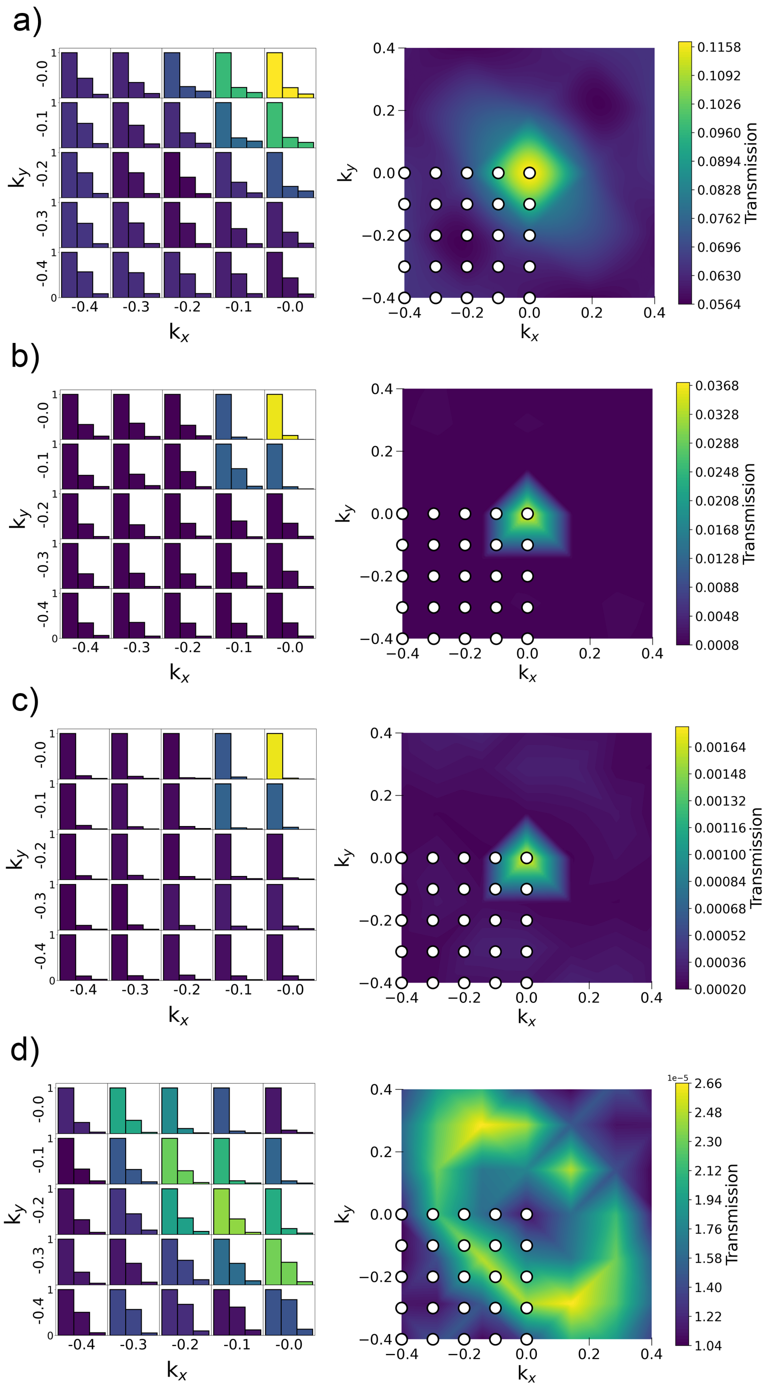

Figure S2: Left: Transmission as a function of k_x and k_y. The white dots indicate the k-points, where the transmission eigenvalues were calculated. Right: The three largest normalized transmission eigenvalues on respective k-points. The molecules investigated are a) (M)-[6]-thiohelicene b) (3S)-3-methylhexane-1,6-diol c) (S)-2-methyl-butanediamide d) (L)-α-Helix with thiolated terminal groups.

S3 Influence of spin-orbit coupling

S3.1 Computational details for two-component calculations including spin–orbit coupling

We performed transmission calculations for 6-helicene with fully relativistic two-component non-collinear spin-orbit coupling (SOC) using the same computational settings as in the one-component treatment. SOC can be described by a second-order expansion of the relativistic Dirac Hamiltonian in terms of the fine structure constant α^6. In QuantumATK, the SOC is included via norm-conserving pseudopotentials. The norm-conserving pseudopotential has two parts, a local part which is coming from the scalar-relativistic pseudopotential and a non-local part which is generated by mapping the solution of Dirac equation that inherently includes SOC. The total pseudopotential is written as

$$\hat{\mathbf{V}} = \hat{\mathbf{V}}^{\mathbf{SR}} + \hat{\mathbf{V}}^{\mathbf{SOC}} = \sum_{l,m} \left[\tilde{V}_l \mathbb{1}_\sigma + \tilde{V}_l^{SOC} \vec{\mathbf{L}} \cdot \vec{\mathbf{S}} \right] |l,m\rangle \langle l,m| \tag{S1}$$

, where m is the spin-angular momentum quantum number and $\mathbb{1}_\sigma$ is a 2×2 unit matrix. The SOC operator can be expressed as,

$$\vec{\mathbf{L}} \cdot \vec{\mathbf{S}} = \frac{1}{2} \begin{pmatrix} \hat{L}_z & \hat{L}_- \\ \hat{L}_+ & \hat{L}_z \end{pmatrix}, \tag{S2}$$

and,

$$\tilde{V}_l = \frac{1}{2l+1} \left[(l+1) V_{l+1/2} + l V_{l-1/2} \right], \tag{S3}$$

$$\tilde{V}_l^{SOC} = \frac{1}{2l+1} \left[V_{l+1/2} + V_{l-1/2} \right]. \tag{S4}$$

Adding these pseudopotentials to the Hamiltonian yields a 2×2 matrix form in spin space,

$$\boldsymbol{H} = \begin{bmatrix} H^{\uparrow\uparrow} & H^{\downarrow\uparrow} \\ H^{\uparrow\downarrow} & H^{\downarrow\downarrow} \end{bmatrix}. \tag{S5}$$

For a detailed derivation, the readers are suggested to follow Refs.[5,6]. The overlap matrix $\boldsymbol{S}$ is calculated from the spinors of the $\boldsymbol{H}$ and has the form,

$$\boldsymbol{S} = \begin{bmatrix} S^{\uparrow\uparrow} & S^{\downarrow\uparrow} \\ S^{\uparrow\downarrow} & S^{\downarrow\downarrow} \end{bmatrix} \tag{S6}$$

The electrode self-energy term $\boldsymbol{\Sigma}_{L/R}$ calculated in Krylov subspace[7,8] is expressed as,

$$\boldsymbol{\Sigma}_{L/R} = \begin{bmatrix} \Sigma^{\uparrow\uparrow}_{L/R} & \Sigma^{\downarrow\uparrow}_{L/R} \\ \Sigma^{\uparrow\downarrow}_{L/R} & \Sigma^{\downarrow\downarrow}_{L/R} \end{bmatrix}. \tag{S7}$$

The equations S5, S6, S7 are included in equation 8 in the main manuscript in order to obtain the two-component Green's function necessary for a two-component transmission function.

S3.2 Results

All transmission eigenvalues shown in the main manuscript were obtained using spinless transport calculations. In the context of CISS, one may ask whether including spin-orbit coupling (SOC) could alter our conclusions. We cannot test for this directly, as there is no implementation for evaluating transmission eigenvalues in combination with SOC in QuantumATK at the moment. As an alternative, we have calculated the transmission spectrum of (M)-[6]-helicene between two gold electrodes (the structure is shown in Fig. 2 in the main manuscript), once with the same methodology as used in the manuscript and once using a two-component, non-collinear spin treatment including spin-orbit coupling (see Figure S3). We found that $T(E)$ is

slightly shifted in energy for the SOC case compared to the non-SOC case, while overall, the transmission remains very similar. This strongly suggests that the transmission eigenvalues will remain similar, too.

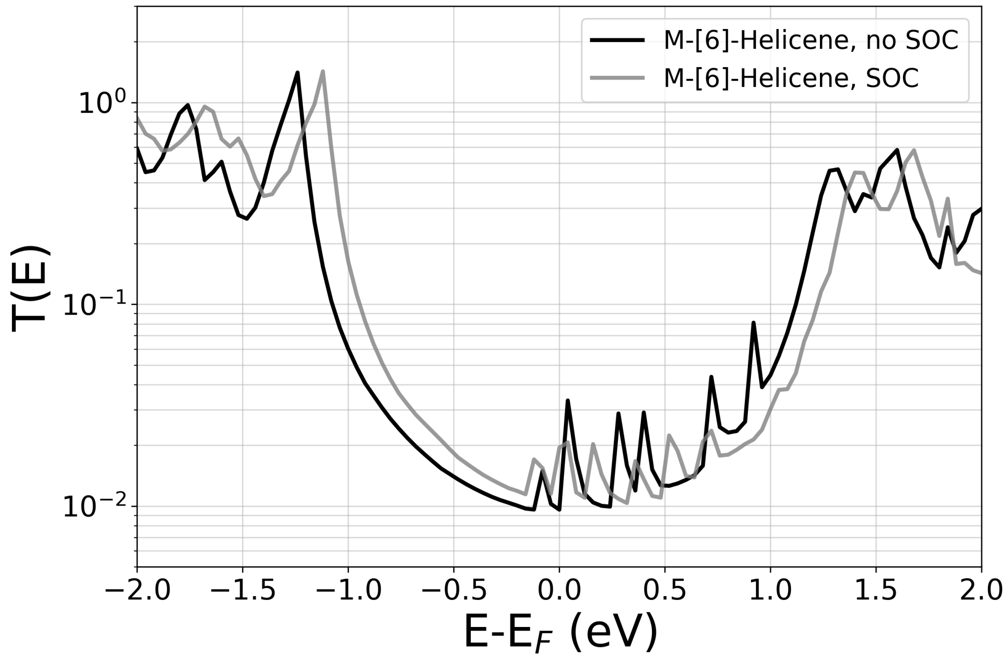

Figure S3: Comparison of the transmission spectra of (M)-[6]-helicene between gold electrodes, obtained from a spinless calculation (black) and from a non-collinear calculation including spin–orbit coupling (grey).

S4 Influence of mirroring the molecule

In this work, we calculated transmission eigenvalues for both enantiomers in such a way that we mirrored the molecule while leaving the electrode underneath unmirrored. To show that this approach does not lead to strongly unrelaxed setups, which could, in turn, change the magnitude of the transmission eigenvalues significantly, we have relaxed the molecular structure of (P)-[6]-Helicene after it was generated by mirroring (M)-[6]-Helicene. The respective transmission eigenvalues for both the unrelaxed and the relaxed cases can be found in the table below.

The additional relaxation step marginally affects the magnitudes of the individual transmission eigenvalues, which justifies the approach to neglect the relaxation step after the mirroring.

Table S1: (6)-[P]-Helicene

unrelaxed	relaxed
3.058425e-03	3.058365e-03
1.040997e-03	1.040993e-03
1.100414e-04	1.100396e-04
2.174387e-05	2.174327e-05

S5 Data for Figures 4 and 5 in the main manuscript

Table S2: Corresponding raw data of Fig. 4 in the main manuscript. The graphic in the manuscript only features the first two columns for visual clarity.

(M)-[6]-Helicene derivative	Functional	λ_1	λ_2	λ_3	λ_4
Pristine	GGA	1.00	1.61×10^{-1}	5.41×10^{-4}	4.94×10^{-5}
	r^2 scan	1.00	2.38×10^{-1}	5.45×10^{-4}	3.73×10^{-5}
	HSE06	1.00	2.78×10^{-1}	469×10^{-4}	3.86×10^{-5}
bis(6-amino)	GGA	1.00	5.71×10^{-2}	4.59×10^{-3}	1.38×10^{-3}
	r^2 scan	1.00	9.71×10^{-2}	2.77×10^{-3}	1.95×10^{-3}
	HSE06	1.00	2.94×10^{-1}	1.83×10^{-2}	8.87×10^{-3}
bis(6-carboxy)	GGA	1.00	3.90×10^{-1}	7.98×10^{-2}	8.66×10^{-3}
	r^2 scan	1.00	3.31×10^{-1}	3.18×10^{-2}	8.27×10^{-3}
	HSE06	1.00	2.42×10^{-1}	1.56×10^{-1}	7.02×10^{-3}
bis(6-bromo)	GGA	1.00	1.07×10^{-1}	3.38×10^{-2}	4.74×10^{-3}
	r^2 scan	1.00	1.23×10^{-1}	3.13×10^{-2}	6.72×10^{-3}
	HSE06	1.00	1.69×10^{-1}	4.98×10^{-2}	8.99×10^{-3}
bis(6-(1,3,4-thiadiazol-2-yl))	GGA	1.00	3.90×10^{-2}	5.18×10^{-3}	1.58×10^{-3}
	r^2 scan	1.00	2.04×10^{-2}	5.96×10^{-3}	9.56×10^{-4}
	HSE06	1.00	2.88×10^{-2}	5.64×10^{-3}	2.45×10^{-3}
bis(6-thio)	GGA	1.00	1.68×10^{-1}	2.73×10^{-2}	1.13×10^{-2}
	r^2 scan	1.00	1.84×10^{-1}	9.67×10^{-2}	3.40×10^{-2}
	HSE06	1.00	2.29×10^{-1}	8.95×10^{-2}	2.96×10^{-2}

Table S3: Corresponding raw data of Fig. 5 in the main manuscript. The graphic in the manuscript only features the first two columns for visual clarity.

Molecule	λ_1	λ_2	λ_3	λ_4
Helicene	1.00	2.79×10^{-1}	4.69×10^{-4}	3.86×10^{-5}
α-Helix	1.00	3.16×10^{-1}	2.66×10^{-2}	9.46×10^{-3}
Lysine	1.00	1.17×10^{-2}	9.08×10^{-5}	7.48×10^{-5}
MH-diol	1.00	9.01×10^{-2}	8.58×10^{-4}	4.01×10^{-4}
MB-diamide	1.00	2.42×10^{-2}	3.09×10^{-3}	1.87×10^{-4}
Tartaric acid	1.00	1.05×10^{-4}	3.05×10^{-6}	4.23×10^{-7}

References

(1) Schlipf, M.; Gygi, F. Optimization algorithm for the generation of ONCV pseudopotentials. *Comput. Phys. Commun.* **2015**, *196*, 36–44.

(2) van Setten, M.; Giantomassi, M.; Bousquet, E.; Verstraete, M.; Hamann, D.; Gonze, X.; Rignanese, G.-M. The PseudoDojo: Training and grading a 85 element optimized norm-conserving pseudopotential table. *Comput. Phys. Commun.* **2018**, *226*, 39–54.

(3) Hartwigsen, C.; Goedecker, S.; Hutter, J. Relativistic separable dual-space Gaussian pseudopotentials from H to Rn. *Phys. Rev. B* **1998**, *58*, 3641–3662.

(4) Blum, V.; Gehrke, R.; Hanke, F.; Havu, P.; Havu, V.; Ren, X.; Reuter, K.; Scheffler, M. Ab initio molecular simulations with numeric atom-centered orbitals. *Comput. Phys. Commun.* **2009**, *180*, 2175–2196.

(5) Smidstrup, S. et al. QuantumATK: an integrated platform of electronic and atomic-scale modelling tools. *J. Phys.: Condens. Matter* **2019**, *32*, 015901.

(6) Fernández-Seivane, L.; Oliveira, M. A.; Sanvito, S.; Ferrer, J. On-site approximation for spin–orbit coupling in linear combination of atomic orbitals density functional methods. *Journal of Physics: Condensed Matter* **2006**, *18*, 7999–8013.

(7) Sørensen, H. H. B.; Hansen, P. C.; Petersen, D. E.; Skelboe, S.; Stokbro, K. Krylov subspace

method for evaluating the self-energy matrices in electron transport calculations. *Physical Review B—Condensed Matter and Materials Physics* **2008**, *77*, 155301.

(8) Sørensen, H. H. B.; Hansen, P. C.; Petersen, D. E.; Skelboe, S.; Stokbro, K. Efficient wave-function matching approach for quantum transport calculations. *Physical Review B—Condensed Matter and Materials Physics* **2009**, *79*, 205322.
